# A genome-wide expression profile of noncoding RNAs in human osteosarcoma cells as they acquire resistance to cisplatin

**DOI:** 10.1007/s12672-021-00441-6

**Published:** 2021-10-20

**Authors:** Harshita Sharma, Divya Niveditha, Rajdeep Chowdhury, Sudeshna Mukherjee, Shibasish Chowdhury

**Affiliations:** Department of Biological Sciences, Birla Institute of Technology and Science (BITS), Pilani Campus, Pilani, Rajasthan 333031 India

**Keywords:** Osteosarcoma, RNA sequencing, Drug resistance, Drug tolerant persister

## Abstract

**Background:**

Recurrence after cisplatin therapy is one of the major hindrances in the management of cancer. This necessitates a deeper understanding of the molecular signatures marking the acquisition of resistance. We therefore modeled the response of osteosarcoma (OS) cells to the first-line chemotherapeutic drug cisplatin. A small population of nondividing cells survived acute cisplatin shock (persisters; OS-P). These cells regained proliferative potential over time re-instating the population again (extended persisters; OS-EP).

**Result:**

In this study, we present the expression profile of noncoding RNAs in untreated OS cells (chemo-naive), OS-P, OS-EP and drug-resistant (OS-R) cells derived from the latter. RNA sequencing was carried out, and thereafter, differential expression (log2-fold ± 1.5; p value ≤ 0.05) of microRNAs (miRNAs) was analyzed in each set. The core set of miRNAs that were uniquely or differentially expressed in each group was identified. Interestingly, we observed that most of each group had their own distinctive set of miRNAs. The miRNAs showing an inverse correlation in expression pattern with mRNAs were further selected, and the key pathways regulated by them were delineated for each group. We observed that pathways such as TNF signaling, autophagy and mitophagy were implicated in multiple groups.

**Conclusion:**

To the best of our knowledge, this is the first study that provides critical information on the variation in the expression pattern of ncRNAs in osteosarcoma cells and the pathways that they might tightly regulate as cells acquire resistance.

**Supplementary Information:**

The online version contains supplementary material available at 10.1007/s12672-021-00441-6.

## Introduction

Osteosarcoma (OS) is the primary malignant tumor of the bone with the highest incidence rate in adults and children [1, 2]. The disease is very aggressive and often manifests as both local and systemic spread, resulting in severe mortality. Additionally, despite aggressive local surgery, OS eventually metastasizes, leading to associated complications and death [3]. This despondency necessitated the development of an alternate arsenal or a belligerent chemotherapeutic regimen for the treatment of OS [4]. Today, pre- and postoperative chemotherapy is an integral part of the OS treatment strategy. However, despite such an aggressive treatment regimen, chemotherapy often fails, resulting in acquired drug resistance and associated recurrence. Therefore, the 5-year survival rate of advanced OS is abysmally lower than 20% [5, 6]. Furthermore, recent efforts to enhance the efficacy of chemotherapy by either drug dose escalation or by modifying drug combinations have been mostly futile, rendering minimal impact on the survival outcome of OS patients [7]. Additionally, to add to desperation, there is hardly any available treatment option for OS that has failed first-line therapy. Hence, the current status of OS pathogenesis and its treatment options perpetually demands an extensive study of molecular alterations, especially those facilitating drug resistance to existing treatment modalities. This might be useful for the identification of novel molecular treatment targets to successfully reinstate drug sensitivity.

In recent years, an increasing number of studies have illustrated the critical role of noncoding RNAs, such as microRNAs (miRNAs) and long noncoding RNAs (lncRNAs), in regulating tumor cell fate. In this context, several studies have portrayed the positive correlation of ncRNA expression with the chemotherapeutic response of tumor cells [8–10]. Therefore, undoubtedly, this emphasizes the importance of these molecules in tumor prognosis. Futuristic studies dissecting the possible contributions of ncRNAs can provide critical insights into the identification of alternate markers that can be exploited for targeting chemotherapy resistance and subsequent relapse.

Structurally, the length of miRNAs varies between 18 and 25 nucleotides, while lncRNAs are predominantly more than 1 kb long. However, both can play a critical role in regulating gene expression at the posttranscriptional level through various mechanisms. This may include direct binding of miRNAs to the target mRNAs, primarily but not exclusively to the 3′-UTR, leading to their translational suppression or degradation. lncRNAs may act as sponges for small RNAs, thus regulating their stability and function [11–15]. However, ncRNA-mediated regulation is both unique and complex; for example, a single miRNA can interact with several regions of one or multiple target mRNAs; similarly, a single mRNA can be regulated by multiple miRNAs. Therefore, understanding the expression profile of ncRNAs and their subsequent complex interaction with target mRNAs can be critical to the design of therapeutic strategies. The fact that recent studies emphasize the growing importance of ncRNAs in chemoresistance allowed us to perform the current study [16, 17].

Herein, we followed a unique strategy to track the molecular changes associated with the sequential process of acquisition of drug resistance in OS cells. The parental HOS cells (referred to as OS) were initially exposed to acute cisplatin shock, allowing the population to crash, setting aside a small subpopulation of nondividing cells (referred to as OS-P) that could sustain toxic drug insult. These cells could survive the drug pressure bottleneck and eventually overcome the restrictive growth state (referred to as OS-EP) and further assimilate the potential to divide and re-establish the tumor population again. OS-EPs were subjected to further drug shock, and the above process was repeated to eventually generate resistant cells (referred to as OS-R). In this study, we performed sequencing of small RNAs (miRNAs) in the abovementioned cell types (OS, OS-P, OS-EP and OS-R). Thereafter, a correlation between miRNA expression and their corresponding mRNA targets was carried out to obtain a holistic idea of transcriptomic alterations associated with each phase. To the best of our knowledge, we are the first group to report sequential alterations of global ncRNAs in OS cells as they attain resistance to the frequently used drug cisplatin.

## Materials and methods

### Cell culture and generation of drug-resistant cell line

The human osteosarcoma cancer cell line HOS-CRL-1543 was procured from NCCS, Pune, India, and cultured at 37 °C and 5% CO_2_ in minimal essential medium (HiMedia) supplemented with 10% fetal bovine serum (Invitrogen). The identity of cell lines was authenticated through STR profiling at Lifecode Technologies Private Limited, New Delhi, India (Project ID: M-1066). Periodic monitoring of the cell line was performed for any contamination. The detailed methodology for the generation of the resistant cells is described elsewhere [18]. Briefly, cells were seeded in 10 cm dishes, grown overnight, and then placed into fresh medium before exposure to a high dose of cisplatin (1 mg/ml) for 2 h. The drug was dissolved in dimethyl sulfoxide (DMSO, SDFCL) and was freshly prepared before each use. While a vast majority of cells died, a very small population of cells survived drug pressure (persisters—OS-P). These cells were slow/nondividing, but over a period of approximately 4 weeks, they re-established their growth potential and revived the population (extended persisters—OS-EP). Thereafter, the cycle of drug treatment followed by revival was repeated four times to generate the cells that showed comparatively increased insensitivity to cisplatin (drug-resistant—OS-R) when compared to untreated parental cells (OS).

### In vitro cytotoxicity assay

In vitro cytotoxicity was performed as described previously by Chowdhury et al. [19]. Briefly, cells were cultured in 96-well plates overnight. The following day, cells were treated with drugs for the desired time. Thereafter, 3-(4,5-dimethylthiazol-2-yl)-2,5-diphenyltetrazolium bromide (MTT) was added to each well and incubated for 4 h. Formazan crystals were solubilized in DMSO, and readings were obtained at 495 nm with a differential filter of 630 nm using an ELISA microplate reader (Start-fax 2100). The percentage of viable cells was calculated using the formula: viability (%) = (mean absorbance value of drug-treated cells)/(mean absorbance value of control)  * 100. A concentration of 0.2% DMSO was found to be nontoxic, was used to dissolve CDDP and was used as a control.

### Total RNA isolation and real-time PCR

Total RNA was isolated from the cultured cells using TRIzol reagent (Invitrogen) according to the manufacturer's protocol. Briefly, four million cells were harvested to extract the total RNA, and the quantity and quality of total RNA were measured using a Nanodrop (GE Healthcare; SimpliNano). Approximately 5 μg of total RNA was outsourced to Bionivid Technology Pvt. Ltd., Bangalore for subsequent RNA sequencing. For complementary DNA (cDNA) synthesis, we used a GeneSure First Strand cDNA Synthesis kit (Genetix) with random hexamers following the manufacturer’s instructions. cDNA templates were amplified for specific genes in a CFX Connect Real-time PCR System (BioRad) and detected using SYBR Green (BioRad). As a control, GAPDH was amplified. The relative RNA expression was calculated using the Livak method [20].

### Non-coding and coding RNA sequencing and analysis

RNA sequencing was performed at Bionivid Technology Pvt. Ltd. The RNA sent for sequencing went through quality control (QC) before subsequent reactions. For sequencing of small RNAs, for each sample, a small RNA library was prepared to start from 1 μg total RNA using TruSeq Small RNA Sample Preparation Kits and protocols (Illumina, San Diego, CA, USA). Briefly, the appropriate cDNA fractions ranging from 18 to 28 nucleotides were separated, purified via 15% denaturing polyacrylamide gel electrophoresis, and then linked to RNA adaptors followed by RT-PCR amplification. Next, the PCR products were further purified on agarose gels to establish libraries. Library quality was checked using a high sensitivity DNA chip (Agilent Technologies, Waldbrunn, Germany). The purified cDNA libraries were used for cluster generation on Illumina's cluster station and sequenced on an Illumina HiSeq 2000 instrument, producing single reads from 49 to 57 base pairs. The small RNA-sequencing raw data files were deposited in NCBI’s Gene Expression Omnibus (GEO) and are accessible by GEO Series accession number GSE86053 (https://www.ncbi.nlm.nih.gov/geo/). Using QC filtered Fastq files, reads that were between 18 and 25 nucleotides were filtered and subjected to BLASTn against the RNA Family database (RFAM) without miRNA sequences. Those unique tags that matched any sequences in the RFAM database at 100% query coverage with less than 2 bp mismatch were filtered. The unmapped unique tags were subjected to the miRProf tool for identification of known miRNAs and the miRCAT tool for identification of novel miRNAs using GrCh38 human genome build. For analysis of miRNA expression, known and novel miRNAs with an eValue of ≤ 0.01 and an average depth of greater than or equal to 5× were considered. Identified known and novel miRNAs with their read counts were subjected to samplewise TPM (transcripts per million) normalization. All the expressed miRNAs were subjected to differential expression analysis using the DESeq package. A fold change of 1.5 and a p value of ≤ 0.05 were used as cut-offs to identify differentially expressed (DE) miRNAs. Unsupervised hierarchical clustering of DE miRNAs was performed using Pearson’s uncentered correlation with the average linkage rule using Cluster 3.0 software and visualized using Java TreeView software. mRNA targets of DE miRNAs were obtained from miRTarBase. A biological analysis network (BAN) of DE target genes, DE miRNAs and GO/pathway harboring them was constructed using BridgeIsland Software from Bionivid Technology Pvt Ltd., Bangalore, India. The resultant file consisting of nodes and edges was imported to Cytoscape V 2.8 to visualize the regulatory network consisting of differentially expressed transcripts, targeting miRNAs and connecting GO/pathways.

The targets of the differentially expressed miRNAs were correlated with mRNA expression data from the same samples. The sequencing of larger RNAs, including mRNAs and lncRNAs, was also performed at Bionivid Technology Pvt. Ltd., Bangalore, and the detailed procedure followed for the same was described earlier [21]. Briefly, RNA was fragmented into 200–300 bp fragments with fragmentation reagent (Ambion, TX, USA), purified by Agencourt RNA Clean beads (Beckman Coulter, MA, USA), and further converted to cDNA through polyA priming. The cDNA libraries were thereafter prepared for sequencing reactions with coverage of 100 nucleotides from each end. Using TopHat (v2.0.11), the sequencing reads were then mapped to the human genome. To further assemble mapped reads against ENSEMBL annotation, Cufflinks (v2.2.1) was used, and the expression level for each transcript was estimated. For differential expression analysis, the expression values were initially converted to FPKM units, and the DE transcripts were identified using CuffDiff. The cut-off for DE was set to a fold change of 1.5 and above, with a p-value of < 0.05. Clustering of DE transcripts was performed using DAVID, and the transcripts were categorized based on Gene Ontology (GO) functional annotations based on three categories: receptor-mediated signaling, intracellular signaling, and functions controlling various other cellular processes. The significantly DE transcripts associated with all three Gene Ontology functional categories were termed ‘key genes’ [22]. Whether the targets of differentially expressed miRNAs, as obtained from miRTarBase, included any of the ‘key genes’ was further analyzed.

## Results

### Overall comparative transcriptomic profile between the four groups of cells

**Fig. 1:**
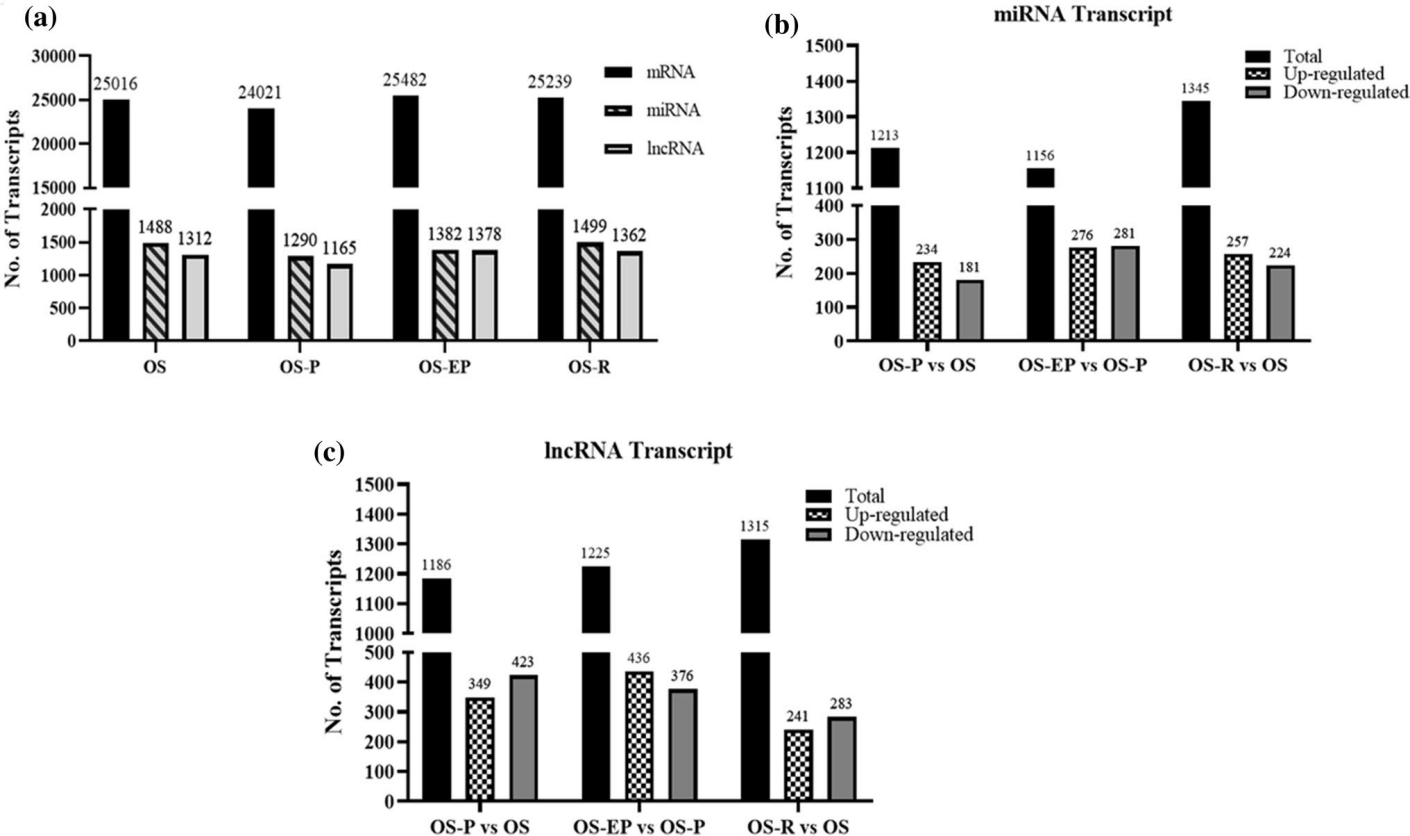
Overall coding and noncoding transcriptomic profile in OS cells. **a** Distribution of different RNA transcripts for untreated parental OS cells (OS), persisters (OS-P), extended persisters (OS-EP) and resistant cells (OS-R). Comparative analysis of **b** miRNA and **c** lncRNA transcripts in the OS-P vs. OS, OS-EP vs. OS-P and OS-R vs. OS sets. The term ‘Total’ indicates the total number of transcripts expressed in the comparison sets, including upregulated, downregulated or neutrally regulated transcripts

Drug resistance has been a major hindrance in the treatment of cancer. Therefore, understanding the molecular signature of cells surviving drug pressure is of utmost clinical relevance. Herein, we analyzed the ncRNA transcriptomic pattern in osteosarcoma cells as they attained resistance to the chemotherapeutic drug cisplatin. Comparative transcriptomic profiling was performed in four groups of cells, which are as follows—OS, untreated parental osteosarcoma cells which consisted of 25,016 mRNA transcripts, 1488 miRNA transcripts and 1312 lncRNA transcripts; OS-P, representing cells surviving cisplatin shock contained 24,021 mRNA transcripts, 1290 miRNA transcripts and 1165 lncRNA transcripts; OS-EP, representing proliferating cells after drug insult comprised of 25,482 mRNA transcripts, 1382 miRNA transcripts and 1378 lncRNA transcripts; OS-R, representing acquired resistant cells consisted of 25,239 mRNA transcripts, 1499 miRNA transcripts and 1362 lncRNA transcripts (Fig. 1a). As evident from the above, the overall transcript pattern for both miRNAs or lncRNAs between the groups did not show a major variation. However, strikingly, an increased number of miRNA transcripts was found to be upregulated (Fig. 1b) in cells under drug shock (OS-P) compared to untreated control (OS), whereas miRNA in other comparative groups showed a trend of downregulation (Fig. 1b). Among the dysregulated lncRNAs, more lncRNA transcripts were downregulated in both OS-P and OS-R cells than in the untreated control (Fig. 1c), whereas in the other two sets, the opposite trend was observed.

### Differential transcriptomic profile between cells surviving cisplatin shock (OS-P) and untreated parental cells (OS)

**Fig. 2:**
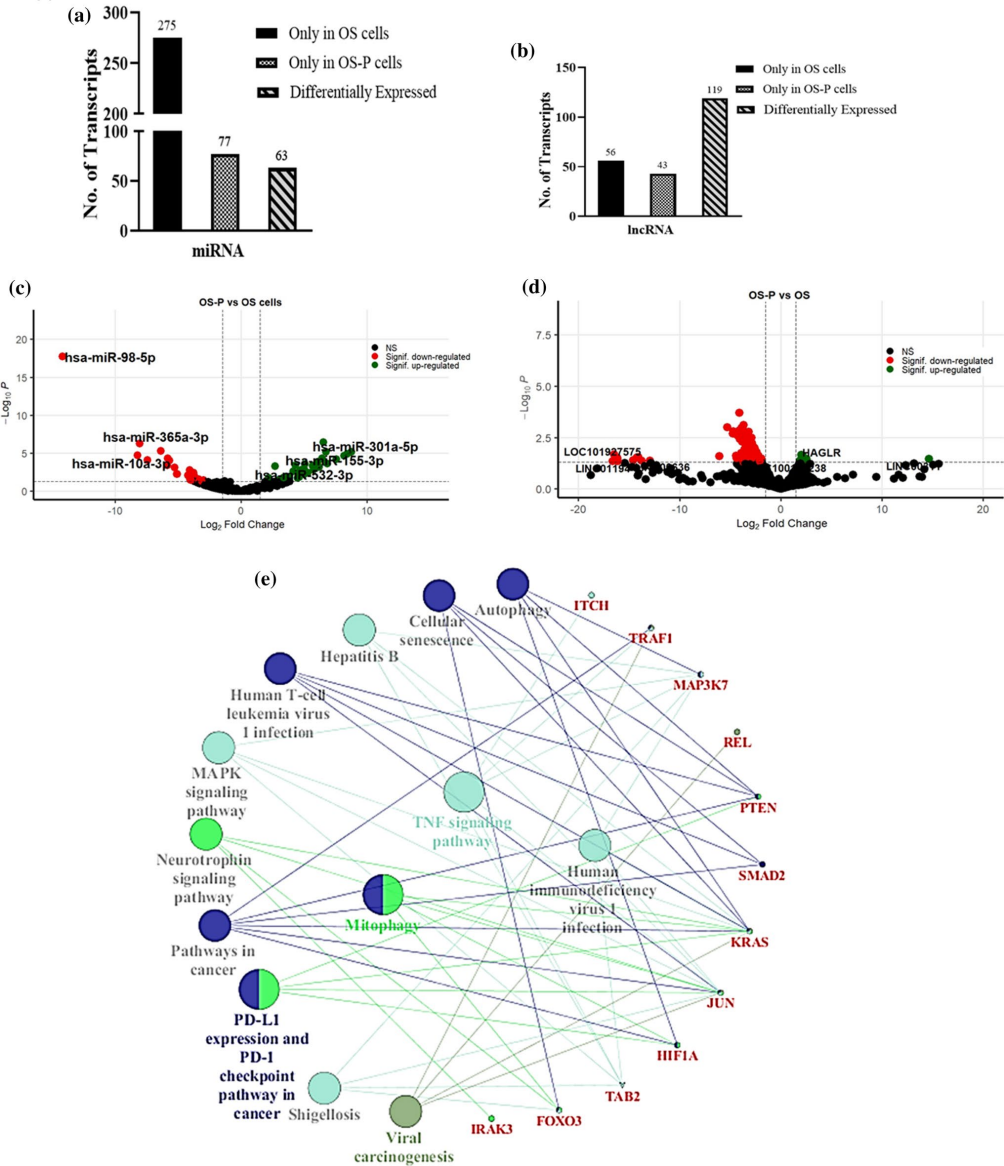
Comparative transcriptomic analysis of OS-P cells with the untreated control (OS). The bar graph represents the **a** miRNA transcript and **b** lncRNA transcript distribution representing the treatment-specific, control-specific and differentially regulated transcripts. In each case, transcripts with a p-value ≤ 0.05 were considered. **c**, **d** Volcano plot representing the upregulated (green dots) and downregulated (red dots). **c** miRNA transcripts and **d** lncRNA transcripts are shown. The names of the top three up- and downregulated miRNA and lncRNA transcripts are mentioned within the plot. Horizontal and vertical dashed lines indicate the significance threshold corresponding to an adjusted p-value of ≤ 0.05 and log2fold change of ± 1.5. **e** Cytoscape network showing key genes and their pathways regulated by significantly dysregulated miRNAs in the OS-P vs. OS set. The key red-labeled genes regulated by miRNAs are densely connected with pathways such as TNF signaling, FOXO signaling, and autophagy

Thereafter, we analyzed the ncRNA expression pattern in each comparative set, starting with the persisters (OS-P) compared to the parental untreated control (OS). The persisters represent a unique population of cells that are sustained and persist under toxic drug insult. The transcriptomic features of these cells were expected to be distinctively supportive of their survival potential under acute drug stress. The miRNA analysis of OS-P with OS revealed that 77 miRNAs were unique and only expressed in OS-P cells, and 63 transcripts showed significant differential expression (p ≤ 0.05; log fold ± 1.5) (Fig. 2a). Similarly, lncRNA expression analysis also showed that 43 lncRNAs were expressed only in OS-P, while 119 transcripts were significantly differentially expressed (Fig. 2b). As evident, the number of differentially expressed lncRNA transcripts was comparatively much higher than that of miRNAs in cells under drug stress (OS-P). Thereafter, a transcript distribution of significantly up- or downregulated miRNAs and lncRNAs was performed and plotted as a volcano plot (Fig. 2c, d). Interestingly, it was observed that a vast majority of lncRNAs were downregulated upon cisplatin shock (OS-P); however, miRNA expression did not follow a similar pattern (Fig. 2c). The total number of significantly differentially regulated miRNAs and lncRNAs is presented in Supplementary Figure 1a. The miRNAs and lncRNAs expressed in persisters only, compared to untreated control are important and might represent molecular signature of the group; the top six miRNA and lncRNA transcripts are represented in Tables 1 and 2, respectively. It has been noticed (Table 1) that the majority of miRNAs that are expressed only in OS-P cells are involved in the repression of cellular proliferation, migration and epithelial–mesenchymal transition (EMT) in different cancer cell types. For example, hsa-miR-374c-5p, hsa-miR-146a-3p, hsa-miR-5100 and hsa-miR-5002-3p are known to inhibit proliferation, metastasis and invasion in breast cancer [23], ovarian cancer [24], pancreatic cancer [25] and gastric cancer cells [26]. Similarly, hsa-miR-1253 has a possible indirect role in tumor suppression through epigenetic silencing in medulloblastoma. Furthermore, lncRNAs such as LINC01488, LRRC75A-AS1, and GNAS-AS1 are reported to be involved in the suppression of cell proliferation and metastasis in hepatocellular carcinoma [27], colorectal sarcoma [28] and lung cancer [29]. A list of the top three up- and downregulated miRNAs and lncRNAs is shown in Tables 3 and 4, respectively. These top dysregulated miRNAs (Table 3) and lncRNAs (Table 4) in OS-P cells are also possibly involved in suppression of cell proliferation, as several earlier studies indicate that the upregulation of hsa-miR-301a-5p [30], hsa-miR-155-3p [31], hsa-miR-532-3p [32], HAGLR [33] and downregulation of hsa-miR-10a-3p [34], hsa-miR-365a-3p [35], LINC01194 [36], LINC00636 [37] are associated with inhibition of cancer growth, proliferation and metastasis in various cancer cell types. Interestingly, miRNAs such as hsa-miR-98-5p and hsa-miR-532-3p (Table 3) are also associated with drug resistance during chemotherapy [38]. Importantly, none of the enlisted unique or differentially regulated transcripts are currently reported to be associated with drug tolerance, thus providing novel insights; however, the association of the majority of these ncRNAs with cancer is already well established.

**Table 1:** Top six treatment-specific miRNA transcripts in OS-P compared to OS cells

miRNA	FPKM	Reported
hsa-miR-374c-5p	152	+
hsa-miR-146a-3p	36	+
hsa-miR-5100	19	+
hsa-miR-1253	6	+
hsa-miR-5002-3p	6	+
hsa-miR-3191-3p	5	+

Fragments per kilobase million (FPKM) value of miRNA transcripts are shown. The miRNA transcripts already reported in cancer-related studies are indicated with the ‘+’ sign, whereas the ‘−’ sign indicates that the specified transcripts are not indicated in any cancer-related studies

**Table 2:** Top six treatment-specific lncRNA transcripts in OS-P compared to OS cells

lncRNA	FPKM	Reported
LINC01488	5	+
LRRC75A-AS1	4	+
CH17-340M24.3	3	−
GNAS-AS1	2	−
LOC101927932	1	−
CDIPT-AS1	1	−

Fragments per kilobase million (FPKM) value is shown. The lncRNA transcripts already reported in cancer-related studies are indicated with the ‘+’ sign, whereas the ‘−’ sign indicates that the specified transcripts are not indicated in any cancer-related studies

**Table 3:** Top three downregulated and upregulated miRNA transcripts in OS-P compared to OS cells

miRNA	log2fold	Reported
hsa-miR-98-5p	− 14.21	+
hsa-miR-10a-3p	− 8.228	+
hsa-miR-365a-3p	− 8.073	+
hsa-miR-301a-5p	8.161	+
hsa-miR-155-3p	7.558	+
hsa-miR-532-3p	6.845	+

In each case, transcripts with a p-value ≤ 0.05 were only considered. The miRNA transcripts already reported in cancer-related studies are indicated with the ‘+’ sign, whereas the ‘−’ sign indicates that the specified transcripts are not indicated in any cancer-related studies

**Table 4:** Top three downregulated and upregulated lncRNA transcripts in OS-P compared to OS cells

lncRNA	log2fold	Reported
LOC101927575	− 16.127	−
LINC01194	− 16.097	+
LINC00636	− 14.578	+
LINC00311	14.617	+
HAGLR	2.554	+
LOC100130238	2.175	−

In each case, transcripts with a p-value ≤ 0.05 were only considered. The lncRNA transcripts already reported in cancer-related studies are indicated with the ‘+’ sign, whereas the ‘−’ sign indicates that the specified transcripts are not indicated in any cancer-related studies

### Analyzing the correlation in expression between ncRNAs and mRNAs in cells surviving cisplatin shock (OS-P)

As mentioned before, unique and differentially expressed ncRNAs can be relevant from the perspective of drug tolerance. To understand their biological function, the probable mRNA targets of the unique and significantly differentially expressed miRNAs were evaluated using mirTarBase. Herein, we analyzed whether the predicted miRNA targets obtained from mirTarBase include any of the ‘key’ mRNA transcripts; earlier, the differentially expressed mRNAs in each group with cellular functions extending into all three Gene Ontology functional annotations—receptor-mediated signaling, cell signaling and other cellular mechanisms were labeled ‘key’ mRNA transcripts. Interestingly, we observed an inverse correlation in the expression pattern between a set of miRNAs and key genes. The top differentially expressed miRNAs showing inverse correlative expression with key mRNAs are shown in Table 5. For example, the miRNAs hsa-miR-27a-3p (log fold: 2.677 and p-value 4.79E−04) and hsa-miR-503-5p (log fold: 8.161 and p-value 2.02E−05), which were upregulated in OS-P, were found to have their targets FOXN2 (log fold − 4.113 and p-value 0.0043) and REL (log fold − 3.809 and p-value 0.013) downregulated, respectively, thus showing an inverse correlation in expression. Furthermore, we observed that the targets of hsa-miR-27a-3p included several ‘key’ genes, indicating a probable role of this miRNA in persistence under drug stress [39–41]. Interestingly, hsa-miR-27a-3p is reported to be associated with cancer cell quiescence, and in corroboration to the above, it has been observed by others and our group that tumor cells exposed to acute drug pressure can enter a transitory nondividing state. To gain further insights into probable pathways regulated by the key genes having inverse correlation with miRNA expression pattern, we analyzed the same using ClueGO, a Cytoscape plugin. The predominant pathways with probable functional significance in drug tolerance emerged as TNF signaling, autophagy, mitophagy, FOXO signaling and others (Fig. 2e). A vast majority of these pathways, such as autophagy or mitophagy, are already reported to be associated with insensitivity to drugs, thus providing critical insights into pathways that can be targeted in cells surviving cisplatin pressure [18, 42, 43]. Our analytical method allowed us to narrow down the numerous differentially expressed transcripts to a list of specific transcripts showing strong correlative expression patterns with ncRNAs, and the corresponding pathway map allowed us to visualize predominant pathways that might be deregulated in tolerant cells.

**Table 5:** List of correlated miRNA and mRNA transcripts observed in OS-P compared to OS

miRNA	log2fold	mRNA	log2fold
hsa-miR-98-5p	− 14.21	TRAF1	14.255
hsa-miR-503-5p	8.161	REL	− 3.809
		TSC22D2	− 2.381
hsa-miR-340-5p	6.523	FOXO3	− 16.165
hsa-miR-139-5p	5.75	JUN	− 4.262
hsa-miR-1273 h-5p	3.706	IRAK3	− 14.471
hsa-miR-30c-2-3p	3.503	ZKSCAN8	− 4.127
hsa-miR-27a-3p	2.677	SMAD2	− 16.102
		NR1D2	− 15.429
		FOXN2	− 4.113

Transcripts with a p-value ≤ 0.05 were considered

### Differential transcriptomic profile between extended persisters (OS-EP) and persisters (OS-P)

**Fig. 3:**
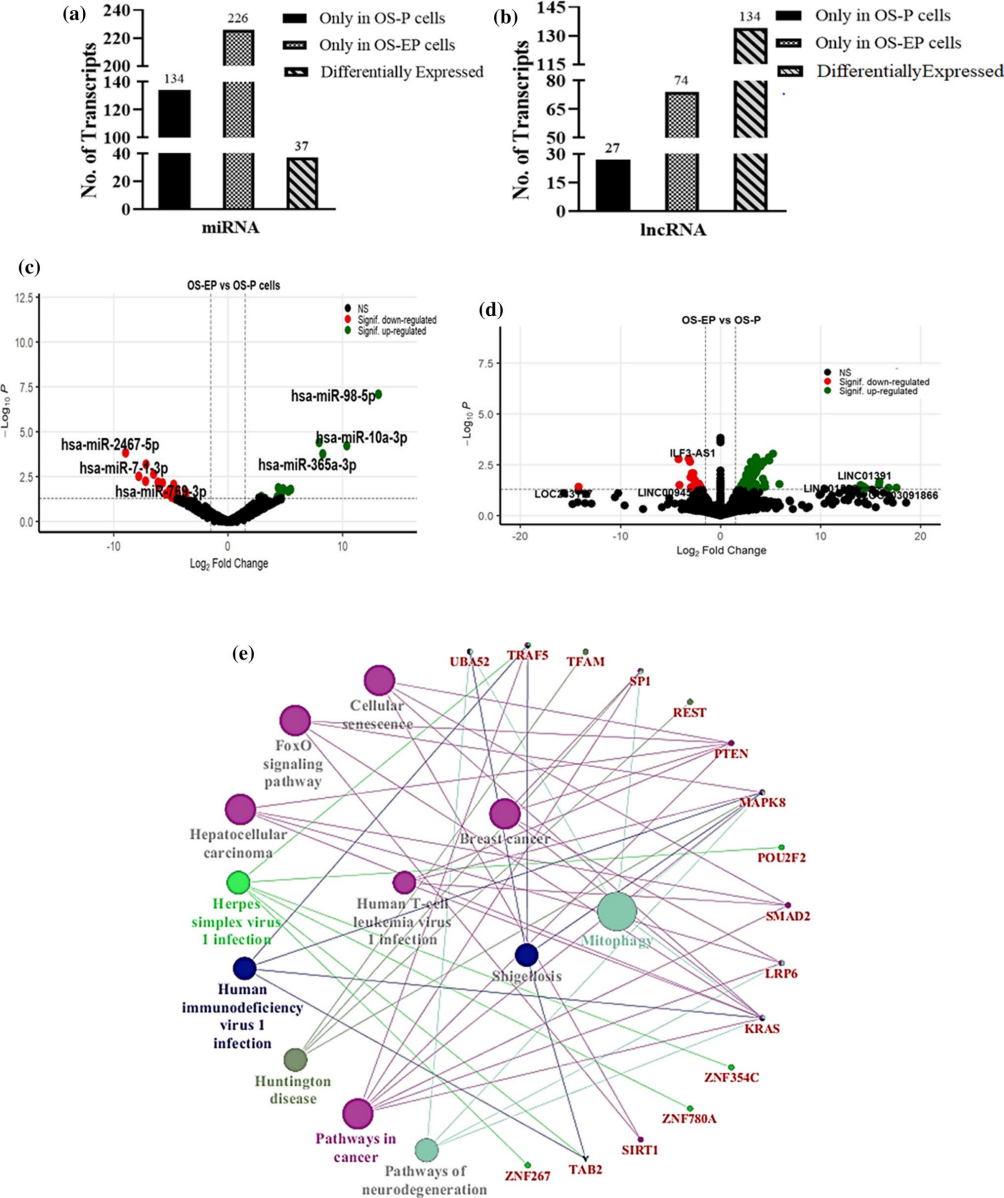
Comparative transcriptomic analysis of cells under cisplatin shock (OS-P) with revived cells (OS-EP). The bar graph represents the **a** miRNA transcript and **b** lncRNA transcript distribution representing the treatment-specific, control-specific and differentially regulated transcripts. In each case, transcripts with a p-value ≤ 0.05 were considered. **c**, **d** Volcano plot representing the upregulated (green dots) and downregulated (red dots). **c** miRNA transcripts. **d** lncRNA transcripts are shown. The names of the top three up- and downregulated miRNA and lncRNA transcripts are mentioned within the plot. Horizontal and vertical dashed lines indicate significance threshold corresponding to an adjusted p-value of ≤ 0.05 and log2fold change of ± 1.5 **e** Cytoscape network showing key genes (labeled in red color) and its pathways regulated by significantly dysregulated miRNAs in OS-EP vs. OS-P set. The key miRNA-regulated genes are densely connected with pathways such as the mTOR signaling pathway, MAPK signaling, autophagy, cellular senescence and apelin signaling

OS-EP represents the group of cells that resumed proliferation after overcoming the growth bottleneck after drug shock. Therefore, this state from the clinical scenario represents the condition when the tumor cells attain proliferative potential surviving a drug insult to regrowth, thus leading to tumor relapse. We expected a significant change in the noncoding RNA expression profile in proliferative OS-EPs compared to growth-restricted cells surviving drug shock. Comparison of expression data between OS-EP and OS-P revealed that approximately 226 miRNA transcripts were expressed only in OS-EP and 37 miRNAs were differentially expressed compared to OS-P cells (Fig. 3a). Similarly, the lncRNA data also showed a total of 74 lncRNAs expressed in only OS-EP, while 134 lncRNAs were differentially expressed (Fig. 3b). The transcript distribution of significantly up- and downregulated miRNAs and lncRNAs was distinctively different from the comparison between OS-P and OS. For instance, the lncRNA transcripts showed an overall upregulation in OS-EP; this was in contrast to what was observed in OS-P compared to the control. A volcano plot for both miRNAs and lncRNAs showing the distribution of differentially regulated transcripts is represented in Fig. 3c, d, respectively. The total number of significantly differentially regulated miRNA and lncRNA transcripts is presented in Supplementary Figure 1b. The top six miRNAs and lncRNAs expressed only in OS-EP are represented in Tables 6 and 7, respectively. miRNAs such as hsa-miR-4697-3p [44], hsa-miR-4758-3p [45], hsa-miR-203b-3p [46] and hsa-miR-3917 [47] have already been reported to have a probable role in drug resistance and relapse in various types of cancer. Importantly, the top six treatment-specific lncRNAs (Table 7), HCCATS-as1, ADGRL3-as1, BCDIN3D-as1, and RALY-as1, are unexplored and are not known for any activity relevant to cancer progression or drug resistance and hence provide valuable insights into molecules that can be relevant for future studies in this context. Furthermore, a list of the top three upregulated and downregulated miRNAs and lncRNAs is shown in Tables 8 and 9, respectively. The majority of dysregulated ncRNAs (hsa-miR-7-1-3p, hsa-miR-769-3p, hsa-miR-98-5p, hsa-miR-10a-3p, hsa-miR-365a-3p, ILF3-AS1, LINC01526 and LINC0139) are known to be involved in cell proliferation, metastasis and drug resistance in various other types of cancers and have not yet been explored in osteosarcoma [34, 38, 48–53].

**Table 6:** Top six treatment-specific miRNA transcripts in OS-EP compared to OS-P cells

miRNA	FPKM	Reported
hsa-miR-3158-5p	126	−
hsa-miR-4697-3p	66	+
hsa-miR-4758-3p	22	+
hsa-miR-6501-5p	19	−
hsa-miR-203b-3p	17	+
hsa-miR-3917	11	+

Fragments per kilobase million (FPKM) value of miRNA transcripts are shown. The miRNA transcripts already reported in cancer-related studies are indicated with the ‘+’ sign, whereas the ‘−’ sign indicates that the specified transcripts are not indicated in any cancer-related studies

**Table 7:** Top six treatment-specific lncRNA transcripts in OS-EP compared to OS-P cells

lncRNA	FPKM	Reported
HCCAT5	3	−
ADGRL3-AS1	3	−
BCDIN3D-AS1	2	−
IGFBP7-AS1	1	−
RALY-AS1	1	−
LINC01483	1	−

Fragments per kilobase million (FPKM) value is shown. ‘−’ sign indicates that the specified transcripts are not indicated in any cancer-related studies

**Table 8:** Top three downregulated and upregulated miRNA transcripts in OS-EP compared to OS-P cells

miRNA	log2fold	Reported
hsa-miR-2467-5p	− 8.946	−
hsa-miR-7-1-3p	− 7.78	+
hsa-miR-769-3p	− 7.172	+
hsa-miR-98-5p	13.139	+
hsa-miR-10a-3p	10.365	+
hsa-miR-365a-3p	8.266	+

In each case, transcripts with a p-value ≤ 0.05 were only considered. The miRNA transcripts already reported in cancer-related studies are indicated with the ‘+’ sign, whereas the ‘−’ sign indicates that the specified transcripts are not indicated in any cancer-related studies

**Table 9:** Top three downregulated and upregulated lncRNA transcripts in OS-EP compared to OS-P cells

lncRNA	log2fold	Reported
LOC283177	− 14.17	+
ILF3-AS1	− 4.185	+
LINC00945	− 4.07	−
LOC103091866	17.595	−
LINC01526	16.805	+
LINC01391	15.876	+

In each case, transcripts with a p-value ≤ 0.05 were only considered. The lncRNA transcripts already reported in cancer-related studies are indicated with the ‘+’ sign, whereas the ‘−’ sign indicates that the specified transcripts are not indicated in any cancer-related studies

### Analyzing the correlation in expression between ncRNAs and mRNAs in persister cells (OS-EP) compared to cells surviving cisplatin shock (OS-P)

Similar to the previous analysis, we thereafter analyzed the probable targets of the unique and differentially expressed miRNAs. The pattern of expression was correlated with the target ‘key gene’ expression in OS-EP. Importantly, we observed that the miRNA hsa-miR-374a-3p (log fold − 7.143 and p-value 0.0006) was downregulated and that its predicted target SMAD2 (log fold 15.783 and p-value 0.0183) was significantly upregulated in OS-EP cells, showing an inverse correlation in expression (Table 10). Conversely, the key gene MAPK8 (log fold 15.694 and p-value 0.0478) was found to be significantly upregulated, while its targeting miRNA hsa-miR-1277-5p (log fold − 4.601 and p-value 0.033) was downregulated. A list of the top significantly differentially expressed mRNA-miRNAs showing inverse correlative expression is presented in Table 10. Furthermore, ClueGO was used to analyze the pathways regulated by these correlated genes. The major functional pathways included mitophagy, cellular senescence and FoxO signaling (Fig. 3e). Importantly, pathways such as TNF signaling, which were implicated in OS-P, were not evident in OS-EP, signifying the dependence of OS-EP cells on unique pathways.

**Table 10:** List of correlated miRNA and mRNA transcripts observed in OS-EP compared to OS-P.

miRNA	log2fold	mRNA	log2fold
hsa-miR-10a-5p	2.935	NACC2	− 2.138
hsa-let-7c-5p	7.96	UBA52	− 2.037
hsa-miR-30d-3p	− 4.452	KRAS	2.609
hsa-miR-1277-5p	− 4.601	LRP6	2.747
		MAPK8	15.694
		TRAF5	17.597
		POU2F2	14.816
hsa-miR-410-3p	− 4.654	REST	14.076
hsa-miR-128-3p	− 4.751	SIRT1	2.45
hsa-miR-374a-3p	− 7.143	SMAD2	15.783

Transcripts with a p-value ≤ 0.05 were considered

### Analyzing the correlation in expression between ncRNAs and mRNAs in resistant cells (OS-R) compared to untreated control (OS)

**Fig. 4:**
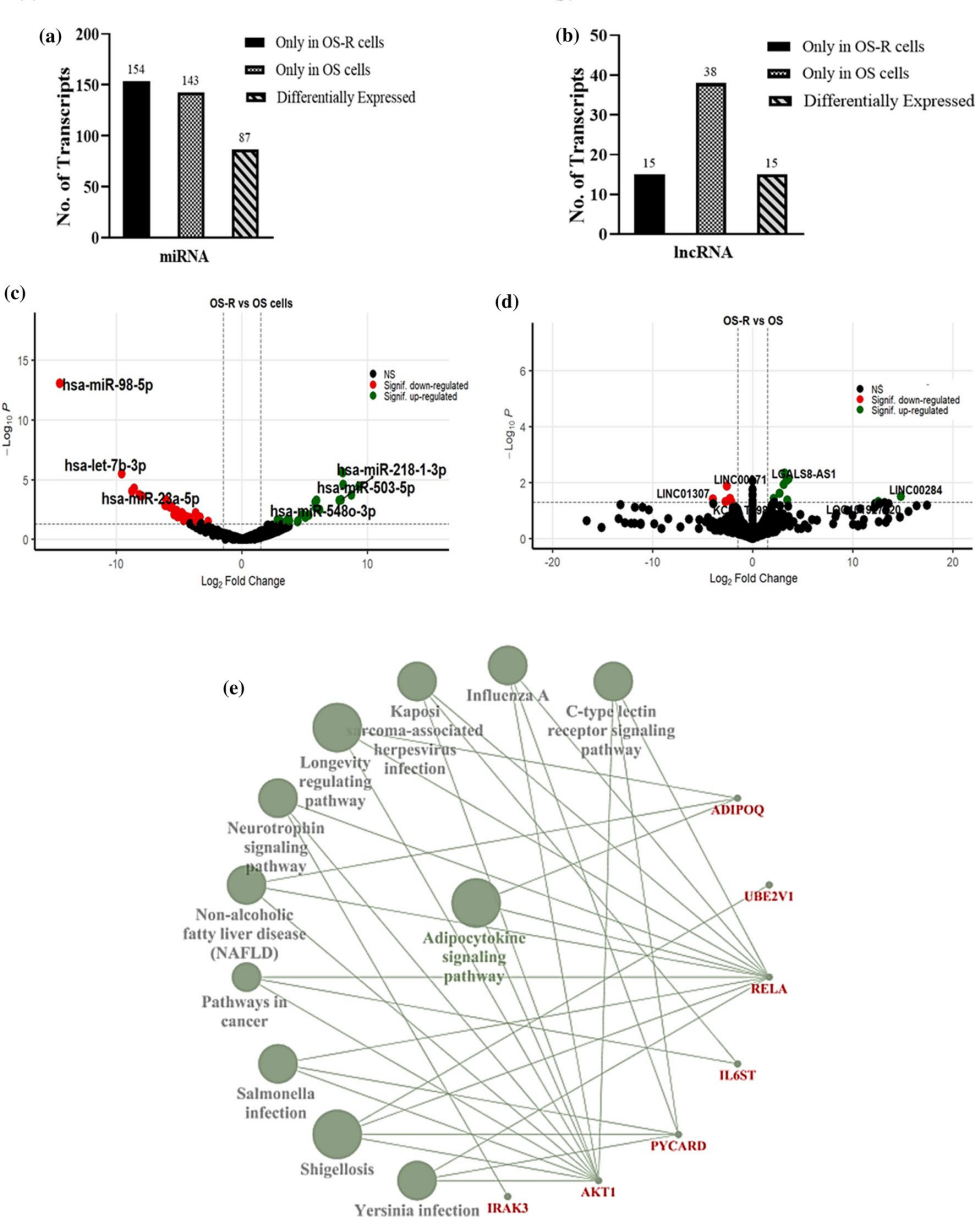
Comparative transcriptomic analysis of drug-resistant cells (OS-R) and untreated controls (OS). The bar graph represents the **a** miRNA transcript and **b** lncRNA transcript distribution representing the treatment-specific, control-specific and differentially regulated transcripts. In each case, transcripts with a p-value ≤ 0.05 were considered. A volcano plot representing the upregulated (green dots) and downregulated (red dots) **c** miRNA transcripts and **d** lncRNA transcripts is shown. The names of the top three up- and downregulated miRNA and lncRNA transcripts are mentioned within the plot. Horizontal and vertical dashed lines indicate significance threshold corresponding to an adjusted p-value of ≤ 0.05 and log2fold change of ± 1.5 **e** Cytoscape network showing key genes (red font) and its pathways regulated by significantly dysregulated miRNAs in OS-R vs. OS set. miRNA-regulated key genes are involved in regulating the adipocytokine signaling pathway and B-cell receptor signaling pathway

The resistant cells (OS-R) derived from OS-EP represent the group of cells that have acquired comparative insensitivity to cisplatin compared to the parental cells. We assume that the ncRNA transcriptomic profile would be distinct in these groups of cells compared to the untreated control (OS) or the cells tolerating acute drug pressure (OS-P). A comparative analysis showed that 154 miRNAs were expressed in OS-R cells only, and 87 miRNAs were differentially expressed (Fig. 4a). Similarly, the lncRNA data analysis showed that 38 lncRNAs were expressed only in OS-R, and 15 were differentially expressed (Fig. 4b). Interestingly, the number of differentially expressed transcripts was comparatively lower in OS-R, suggesting that the cells post attainment of resistance depend on restricted yet specific transcripts to maintain the acquired status. Earlier analysis has also shown that the differential regulation of mRNA expression in OS-R cells is much less drastic with fewer ‘key’ genes than the cells under acute drug shock. A volcano plot representing the distribution of differentially regulated miRNAs and lncRNAs is represented in Fig. 4c, d, respectively. The numerical distribution of differentially regulated miRNAs and lncRNAs is presented in Supplementary Figure 1c. A list of the top treatment-specific and differentially expressed miRNAs and lncRNAs is provided in Tables 11, 12, 13 and 14. Among the OS-R-specific miRNAs (Table 11), we noted miRNAs such as hsa-miR-8065 and hsa-miR-4797-3p that were unexplored in the context of drug resistance, while hsa-miR-374c-5p, miR-6810-5p and hsa-miR-135a-3p have already been reported to have functions in resistance to chemotherapy in ovarian [54], lung and breast cancer [55, 56]. Interestingly, hsa-miR-146a-3p is reported as a circulating miRNA marker for the prognosis of refractory epilepsy. Among the lncRNAs expressed in OS-R only (Table 12), LUCAT1 was found to modulate cancer cell viability and chemotherapy response in various cancer types, including lung, thyroid and CRC [57–59]. Dysregulated miRNAs such as hsa-miR-98-5p [38], hsa-let-7b-3p [60], hsa-miR-23a-5p [61], and hsa-miR-218-1-3p [62] are reported to be involved in cisplatin-induced chemoresistance, whereas miR-503-5p [63] and hsa-miR-548o-3p are known to be involved in cancer progression (Table 13). Importantly, we observed that the majority of dysregulated lncRNAs in OS-R (Table 14) are unique and have not been previously reported for drug resistance, thus providing a pool of markers that can be explored in the future for their putative functional role in osteosarcoma and cisplatin resistance. Interestingly, LINC00284, which was highly upregulated in OS-R, was previously correlated with the promotion of cellular proliferation, invasion, migration, and angiogenesis and the negative regulation of apoptosis-related pathways in a recent study, indicating a probable role that it might play in cisplatin resistance in OS cells [64]. Furthermore, despite the limited number of miRNAs being upregulated, we observed that hsa-miR-30c-1-3p, hsa-miR-19a-3p, hsa-miR-5582-3p and hsa-miR-615-3p showed inverse correlations in expression with key genes that were expressed in OS-R cells. For example, hsa-miR-19a-3p (log fold − 4.429 and p-value 0.0122) showed downregulation, while its predicted key gene target AKT1 was only expressed in OS-R cells (p-value 0.07). The expression of AKT1 was validated by real-time PCR, which showed a significant upregulation in OS-R (Supplementary Figure 1d). Furthermore, the pathways regulated by the correlative key genes in OS-R were analyzed, including those related to adipocytokine signaling, neurotrophin signaling and others that are known to be relevant to cancer (Fig. 4e). To further validate our findings, we compared the OS-R expression data with two independent expression profile datasets on drug-resistant osteosarcoma cells obtained from GEO. Although the experimental design and OS cells used between the studies varied, quite a few genes showed a similar pattern of differential expression, with our data having a p value significance (< 0.05). Few examples of such genes with a correlative expression pattern included LINC00589, APTX, CDH13C and CYB561 (OS-R compared to GSE16089) and SET, LDHA, and DUSP6 (OS-R compared to GSE3362), which might be of key relevance in the context of drug resistance. Additionally, to understand how drug-tolerant persisters (OS-P) differ from subsequently derived resistant OS-R cells, we compared their transcriptomic profiles. A heatmap comparing the expression pattern of miRNAs indicates that the transcriptional profiles in these two sets are quite different (Supplementary Figure 1e). It is evident that a substantial number of miRNAs are uniquely expressed in each set, suggesting a differential transcriptional program primarily active in OS-R and OS-P.

**Table 11:** Top six treatment-specific miRNA transcripts in OS-R compared to OS cells

miRNA	FPKM	Reported
hsa-miR-8065	1597	−
hsa-miR-374c-5p	515	+
hsa-miR-146a-3p	29	+
hsa-miR-6810-5p	16	+
hsa-miR-4797-3p	15	−
hsa-miR-135a-3p	13	+

Fragments per kilobase million (FPKM) value of miRNA transcripts are shown. The miRNA transcripts already reported in cancer-related studies are indicated with the ‘+’ sign, whereas the ‘−’ sign indicates that the specified transcripts are not indicated in any cancer-related studies

**Table 12:** Top six treatment-specific lncRNA transcripts in OS-R compared to OS cells

lncRNA	FPKM	Reported
LRRC75A-AS1	5	+
VLDLR-AS1	5	−
LOC284865	2	−
PIK3CD-AS2	1	+
LURAP1 L-AS1	1	−
LUCAT1	1	+

Fragments per kilobase million (FPKM) value of miRNA transcripts are shown. The lncRNA transcripts already reported in cancer-related studies are indicated with the ‘+’ sign, whereas the ‘−’ sign indicates that the specified transcripts are not indicated in any cancer-related studies

**Table 13:** Top three downregulated and upregulated miRNA transcripts in OS-R compared to OS cells

miRNA	log2fold	Reported
hsa-miR-98-5p	− 14.501	+
hsa-let-7b-3p	− 9.579	+
hsa-miR-23a-5p	− 8.584	+
hsa-miR-503-5p	8.721	+
hsa-miR-218-1-3p	7.918	+
hsa-miR-548o-3p	7.781	−

In each case, transcripts with a p-value ≤ 0.05 were only considered. The miRNA transcripts already reported in cancer-related studies are indicated with the ‘+’ sign, whereas the ‘−’ sign indicates that the specified transcripts are not indicated in any cancer-related studies

**Table 14:** Top three downregulated and upregulated lncRNA transcripts in OS-R compared to OS cells

lncRNA	log2fold	Reported
LINC01307	− 3.975	−
LINC00971	− 2.576	−
KCCAT198	− 2.696	−
LINC00284	14.838	+
LOC101927020	12.567	−
LGALS8-AS1	3.375	−

In each case, transcripts with a p-value ≤ 0.05 were only considered. The lncRNA transcripts already reported in cancer-related studies are indicated with the ‘+’ sign, whereas the ‘−’ sign indicates that the specified transcripts are not indicated in any cancer-related studies

### Analyzing the correlation in expression between lncRNA, miRNA and mRNA expression in all the groups

**Fig. 5:**
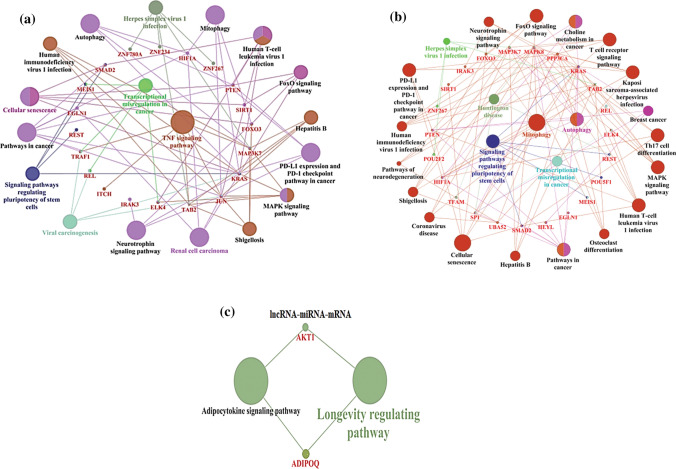
**a** Cytoscape network of the lncRNA-miRNA axis regulating key mRNA genes in OS-P vs. OS cells. **b** Cytoscape network of lncRNA-miRNA regulating key mRNA genes in the OS-EP vs. OS-P set. **c** Cytoscape network of lncRNA-miRNA regulating key mRNA genes in OS-R vs. OS cells

lncRNAs can often regulate gene expression by acting as sponges for miRNAs; therefore, understanding their expression in association with miRNAs and mRNAs has become critical. To obtain a correlative understanding of the expression between lncRNA-miRNA and mRNA patterns, we further analyzed the differentially regulated miRNAs with potential binding affinity with the significantly deregulated lncRNAs in each group using DIANA-LncBase v3. Thereafter, the key genes targeted by these shortlisted miRNAs were analyzed. The top differentially expressed lncRNAs in OS-P compared to OS showing correlative expression with miRNAs and other mRNAs are shown in Table 15. The correlative ‘key’ genes were further taken as input to construct a pathway map that highlighted pathways such as TNF signaling, mitophagy and autophagy, which might be tightly regulated in persisters through a correlative expression network (Fig. 5a).

**Table 15:** The significantly differentially expressed lncRNAs in OS-P compared to OS showing correlative expression with miRNAs and further mRNAs

lncRNA	log2fold	p-value	miRNA	log2fold	p-value	mRNA	log2fold	p-value
HIPK1-AS1	− 14.223	0.0296	hsa-miR-139-5p.1	5.758	0.001547	JUN	− 4.262	0.00235
CKMT2-AS1	− 14.052	0.02785	hsa-miR-27a-3p.1	2.677	0.000479	NR1D2	− 15.429	0.04325
LINC00674	− 13.758	0.0433	hsa-miR-139-5p.2	5.758	0.001547	JUN	− 4.262	0.00235
LINC00641	− 4.365	0.03165	hsa-miR-27a-3p.2	2.677	0.000479	SMAD2	− 16.102	0.0179
SOX21-AS1	− 3.995	0.0032	hsa-miR-27a-3p.3	2.677	0.000479	NR1D2	− 15.429	0.04325
MALAT1	− 3.835	0.00505	hsa-miR-27a-3p.4	2.677	0.000479	SMAD2	− 16.102	0.0179
LINC01554	− 3.236	0.00235	hsa-miR-27a-3p.5	2.677	0.000479	SMAD2	− 16.102	0.0179
LUCAT1	− 3.231	0.0153	hsa-miR-27a-3p.6	2.677	0.000479	SMAD2	− 16.102	0.0179
OIP5-AS1	− 2.979	0.0073	hsa-miR-139-5p.3	5.758	0.001547	JUN	− 4.262	0.00235
LINC01278	− 2.274	0.0334	hsa-miR-139-5p.4	5.758	0.001547	JUN	− 4.262	0.00235

Similarly, the top differentially expressed lncRNAs showing correlative expression with miRNAs and mRNAs in OS-EP compared to OS-P are shown in Table 16. The key mRNAs when taken as input to construct a pathway map showed signaling pathways regulating stemness, transcriptional misregulation in cancer, mitophagy, cellular senescence and FOXO signaling (Fig. 5b). In this analysis, we also found that miRNAs implicated in stemness and cellular differentiation, such as hsa-let-7c-5p, showed a correlation with lncRNA and mRNA expression patterns.

**Table 16:** The significantly differentially expressed lncRNAs in OS-EP compared to OS-P showing correlative expression with miRNAs and mRNAs

lncRNA	log2fold	P value	miRNA	log2fold	P value	mRNA	log2fold	P value
FBXL19-AS1	− 2.116	0.0296	hsa-let-7c-5p	7.96	3.91E−05	UBA52	− 2.037	0.0331
LINC00240	− 1.98	0.04095	hsa-let-7c-5p	7.96	3.91E−05	UBA52	− 2.037	0.0331
LINC00865	1.941	0.04645	hsa-miR-142-5p	4.541	0.040141	SIVA1	− 2.406	0.01755
LINC00674	13.851	0.04455	hsa-miR-128-3p	− 4.751	0.008817	SMAD2	15.783	0.01835
LINC00865	1.941	0.04645	hsa-miR-19b-3p	− 6.497	0.002331	PTEN	2.736	0.0079

Finally, the list of lncRNA–miRNA–mRNA showing inverse correlative expression in OS-R compared to OS is presented in Table 17. Herein, we observed that lncRNAs LGALS8-AS1 (log fold 3.375 and p-value 0.008), hsa-miR-19a-3p (log fold − 4.429 and p-value 0.012) and AKT1 (log fold 0 and p-value 0.07) and LINC00284 (log fold 14.838 and p-value 0.003), hsa-miR-19a-3p (log fold − 4.429 and p-value 0.012) and AKT1 (log fold 0 and p-value 0.07) showed correlations. The above lncRNAs might be critical to the acquisition of resistance. Cytoscape analysis of these targets showed enriched pathways such as adipocyte signaling and longevity regulating pathways (Fig. 5c).

**Table 17:** The significantly differentially expressed lncRNAs in OS-R compared to OS showing correlative expression with miRNAs and mRNAs

lncRNA	log2fold	P value	miRNA	log2fold	P value	mRNA	log2fold	P value
LGALS8-AS1	3.375	0.00845	hsa-miR-19a-3p	− 4.429	0.012264	AKT1	0	0.07305
LINC00284	14.838	0.03145	hsa-miR-19a-3p	− 4.429	0.012264	AKT1	0	0.07305
LINC00589	3.105	0.0115	hsa-miR-7-5p	− 1.584	0.667092	RELA	0	0.14075

## Discussion

Noncoding RNAs (ncRNAs) are no longer regarded as bystanders; rather, they are found to have a profound influence in regulating tumor development and therapy resistance through a plethora of [26, 65, 66]. ncRNAs can tightly and dynamically regulate the processing of protein-coding genes during the process of tumor development, thus having a deep impact on tumor cell transcriptional and translational control. Given the importance of ncRNAs in tumor biology, a deeper understanding of their regulation and function might facilitate the development of future cancer therapeutics. In this regard, the dynamic expression pattern of noncoding RNAs, including miRNAs and lncRNAs, as tumor cells attain drug resistance, and the correlative expression of their putative targets has been poorly characterized. In this study, we performed next-generation sequencing followed by extensive in silico analysis to obtain the correlative expression pattern of ncRNAs and mRNAs from cells at different stages during the process of acquisition of drug resistance. The ncRNAs significantly deregulated at each stage were identified, their putative mRNA targets were predicted, and the set of transcripts showing correlative expression patterns with respect to the mRNA expression patterns were short-listed. Finally, a ncRNA-mRNA correlative expression network was constructed, and the pathways regulated by the correlated transcripts were identified. Our study, for the first time, provides valuable information on how the ncRNA expression profile dynamically changes with respect to the mRNA expression pattern in osteosarcoma cells as they acquire resistance to the widely used drug cisplatin. Additionally, we provide a holistic overview of the ncRNA signature and putative pathways regulated by them representing each stage during the process of acquisition of resistance by tumor cells.

Interestingly, our results reveal that cells initially exposed to high cisplatin shock show a decreased number of upregulated mRNAs or lncRNAs but, in contrast, show an increased level of upregulated miRNA transcripts, indicating probable miRNA-mediated regulation under acute drug stress. Further analysis showed that the majority of the dysregulated miRNAs in OS-P are reported to be involved in the suppression of cancer growth, proliferation and metastasis, suggesting a miRNA-mediated attainment of a transitory nondividing state—a strategy to survive acute drug insult. Similarly, epigenetically driven sparsely diving cells, labeled ‘tolerant’ cells, have been observed by multiple research groups; however, the miRNA signature of these cells is poorly understood. Interestingly, miRNAs such as miR-27a-3p, known for its role in regulating cancer stemness and implicated in the pathogenesis of several solid tumors, were found to be significantly upregulated in the persisters, indicating miRNA-mediated induction of stemness as a strategy to survive and result in subsequent recurrence [40]. Importantly, the predicted targets of miR-27a-3p included genes such as SMAD2, FOXN2 and NR1D2, which showed a correlatively significant downregulation in OS-P cells. The key pathways derived from mRNAs showing inverse correlative expression with ncRNAs included important pathways such as TNF signaling, autophagy and mitophagy. Currently, there is abundant literature that implicates the role of these cellular homeostatic processes in the survival of tumor cells under drug stress. Thus, our analysis provides cues about molecular pathways that can be targeted to eradicate the reservoir of cells surviving chemotherapy stress with the potential to contribute to relapse and tumor resistance.

Interestingly, as persister cells regain proliferative potential, understandably, the expression profile of ncRNAs changes distinctively as well. A contrasting expression pattern of miRNAs and mRNAs was observed between the cells that attained proliferative potential after drug shock (OS-EP) compared to OS-P. Here, more miRNA transcripts were downregulated, while there was an increase in upregulated mRNA transcripts. Importantly, miRNA hsa-miR-1277-5p emerged as having many key mRNA genes representing the OS-EP signature as its target; however, there are limited studies in the literature evaluating the functional role of this miRNA. Thus, our study not only provides information on pathways already implicated in chemoresistance but also contributes to the identification of novel molecules that can be investigated in this context. The other miRNAs that were found to be deregulated included the well-studied hsa-let-7c-5p, which is known to have a role in stemness and is considered an important biomarker in cancer. Finally, it is worth mentioning that the number of differentially regulated transcripts decreased drastically as cells attained resistance (OS-R); however, they overexpressed specific genes, such as Akt1, that showed correlative expression with ncRNAs. Herein, interestingly, we observed that the miRNA hsa-miR-19a-3p is downregulated in OS-R, and the existing literature suggests that it is often associated with cancer progression; importantly, one of its targets is AKT, which is extensively implicated in cancer and drug resistance. Analysis of pathways deregulated in OS-R cells highlighted the importance of adipocytokine signaling; however, its connection with drug resistance remains to be elucidated. Our study thus suggests that OS-R cells adapt unique strategies to maintain resistance that significantly differ from cells at early stages of acquisition of resistance, and further research in this direction can provide novel insights into ways to tam chemoresistance in osteosarcoma.

## Conclusion

Overall, our study provides critical insights into the dynamic expression pattern of ncRNAs in osteosarcoma cells as they attain resistance to cisplatin. We also identified a pool of ncRNAs that can be validated as signature molecules through future studies and further provided information on putative mRNA transcripts that show correlative expression with ncRNAs. We believe that the holistic expression data obtained from this study will definitely serve as a useful platform for prospective future biomarker studies that can be of clinical relevance and offer better strategies for efficient diagnosis and therapy of OS.

## Supplementary Information

Below is the link to the electronic supplementary material.

Supplementary file 1. Figure 1 **a** Total number of significantly dysregulated miRNA and lncRNA transcripts in OS-P cells compared to untreated controls (OS). Transcripts with a fold change of ± 1.5 and a p-value of ≤ 0.05 were considered significantly dysregulated. **b** Total number of significantly dysregulated miRNA and lncRNA transcripts in OS-EP cells compared to OS-P. Transcripts with a fold change of ± 1.5 and p-value of ≤ 0.05 were considered significantly dysregulated. **c** Total number of significantly dysregulated miRNA and lncRNA transcripts in OS-R cells compared to OS. Transcripts with a fold change of ± 1.5 and a p-value of ≤ 0.05 were considered significantly dysregulated. **d** Bar graph showing the fold change in the expression level of AKT1 as analyzed by qRT-PCR. The symbol * indicates a statistically significant difference.

Supplementary file 2. Figure 1 **e** Heatmap representing the comparative expression profile of miRNAs deregulated in OS-P and OS-R compared to OS. The differentially regulated miRNAs with a p value cut off of ≤ 0.05 were considered.

## Data Availability

The datasets used and/or analyzed during the current study are available from the corresponding author on reasonable request.
