# Supplementary figures and images for: A genome-wide expression profile of noncoding RNAs in human osteosarcoma cells as they acquire resistance to cisplatin
Source: Discov Oncol. 2021 Oct 20;12:43. doi: 10.1007/s12672-021-00441-6 (PMC8777531; doi:10.1007/s12672-021-00441-6)

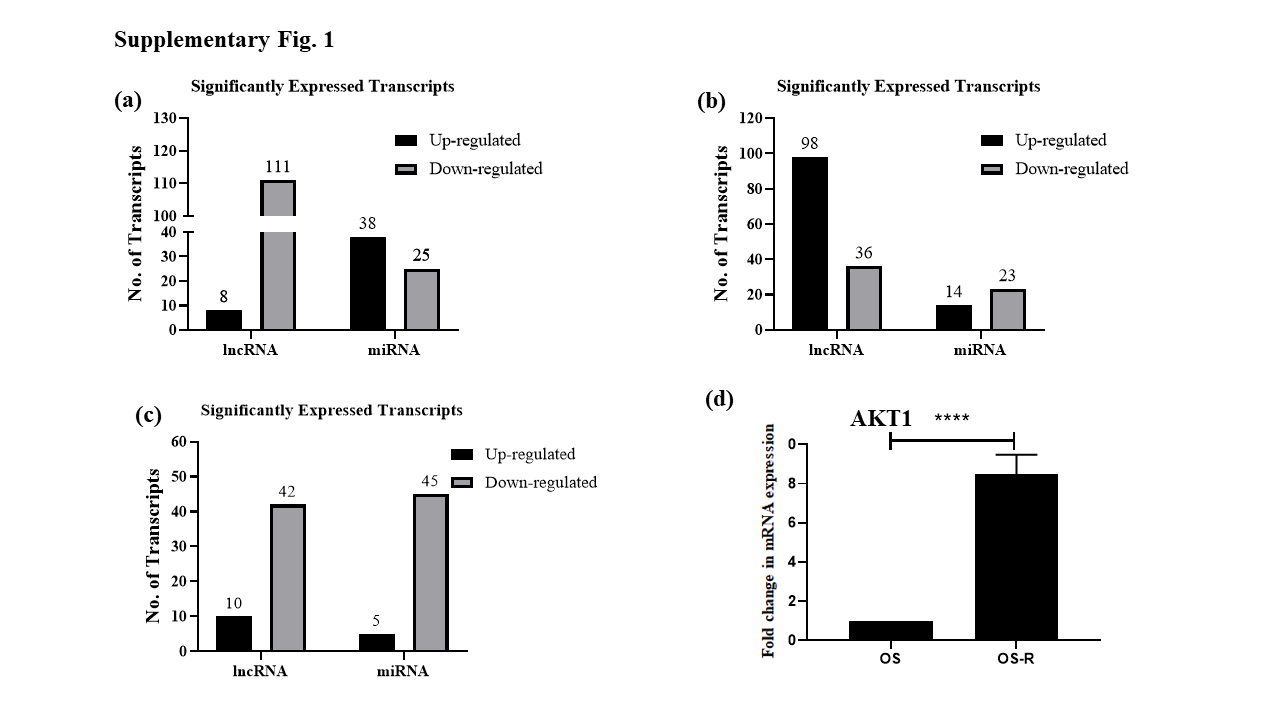

Supplement: Supplementary file 1 — Figure 1 a Total number of significantly dysregulated miRNA and lncRNA transcripts in OS-P cells compared to untreated controls (OS). Transcripts with a fold change of ± 1.5 and a p-value of ≤ 0.05 were considered significantly dysregulated. b Total number of significantly dysregulated miRNA and lncRNA transcripts in OS-EP cells compared to OS-P. Transcripts with a fold change of ± 1.5 and p-value of ≤ 0.05 were considered significantly dysregulated. c Total number of significantly dysregulated miRNA and lncRNA transcripts in OS-R cells compared to OS. Transcripts with a fold change of ± 1.5 and a p-value of ≤ 0.05 were considered significantly dysregulated. d Bar graph showing the fold change in the expression level of AKT1 as analyzed by qRT-PCR. The symbol * indicates a statistically significant difference. [file 12672_2021_441_MOESM1_ESM.tif]

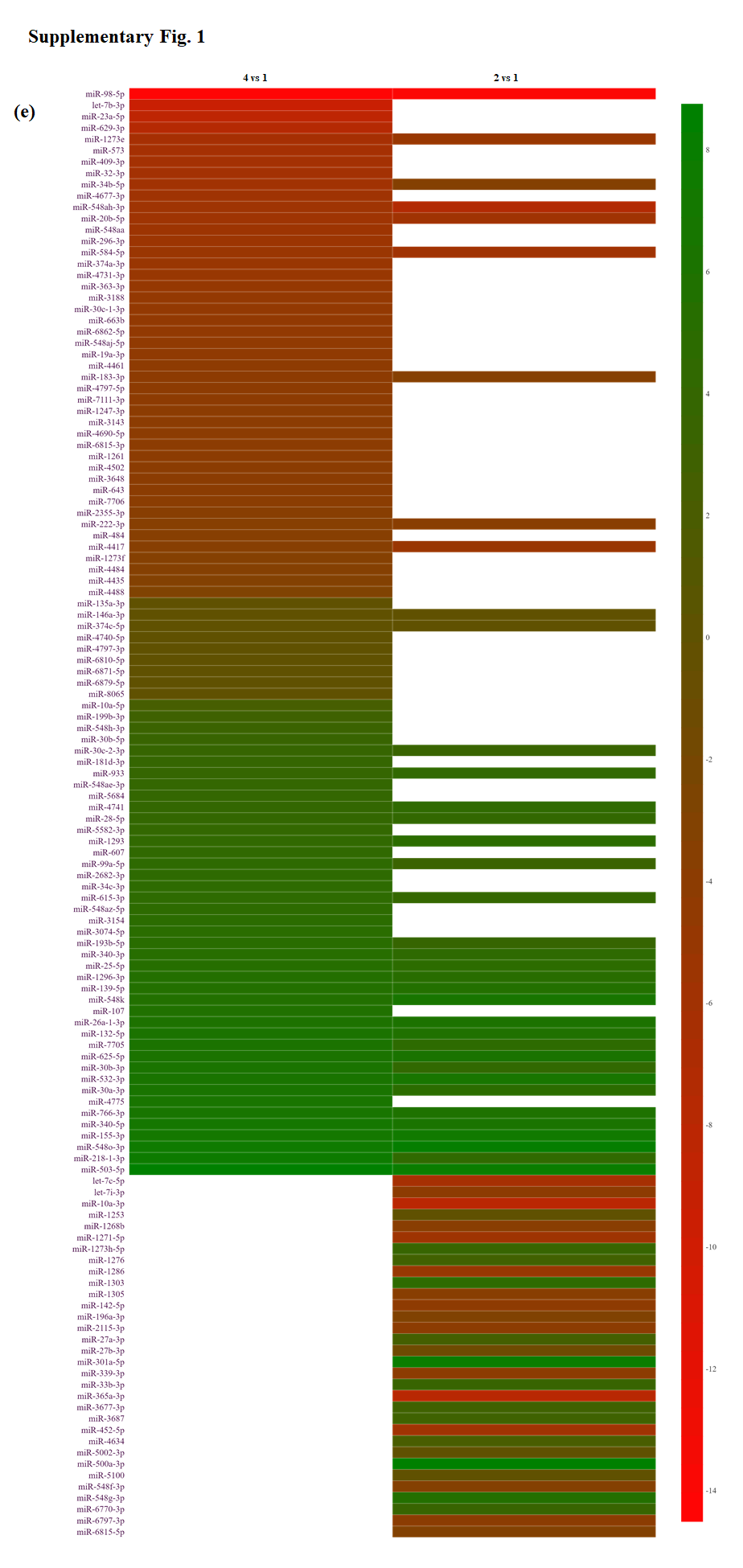

Supplement: Supplementary file 2 — Figure 1 e Heatmap representing the comparative expression profile of miRNAs deregulated in OS-P and OS-R compared to OS. The differentially regulated miRNAs with a p value cut off of ≤ 0.05 were considered. [file 12672_2021_441_MOESM2_ESM.tif]
